# Mannose-Binding Lectin 2 Gene and Risk of Adult Glioma

**DOI:** 10.1371/journal.pone.0061117

**Published:** 2013-04-18

**Authors:** Dominique S. Michaud, Afshan Siddiq, David G. Cox, Danielle M. Backes, Federico C. F. Calboli, Michael E. Sughrue, J. Michael Gaziano, Jing Ma, Meir Stampfer, Shelley S. Tworoger, David J. Hunter, Carlos A. Camargo, Andrew T. Parsa

**Affiliations:** 1 Department of Epidemiology, Brown Public Health, Brown University, Providence, Rhode Island, United States of America; 2 School of Public Health, Faculty of Medicine, Imperial College London, London, United Kingdom; 3 Lyon Cancer Research Center, INSERM U1052/CNRS UMR 5286/UCBL1, Lyon, France; 4 Department of Neurological Surgery, University of Oklahoma, Norma, Oklahoma, United States of America; 5 Division of Preventive Medicine, Brigham and Women’s Hospital, Boston, Massachusetts, United States of America; 6 Channing Division of Network Medicine, Department of Medicine, Brigham and Women’s Hospital and Harvard Medical School, Boston, Massachusetts, United States of America; 7 Department of Epidemiology, Harvard School of Public Health, Boston, Massachusetts, United States of America; 8 Department of Nutrition, Harvard School of Public Health, Boston, Massachusetts, United States of America; 9 Department of Emergency Medicine, Massachusetts General Hospital, Boston, Massachusetts, United States of America; 10 Department of Neurological Surgery, University of California San Francisco, San Francisco, California, United States of America; Vanderbilt University, United States of America

## Abstract

**Background and Aims:**

The immune system is likely to play a key role in the etiology of gliomas. Genetic polymorphisms in the mannose-binding lectin gene, a key activator in the lectin complement pathway, have been associated with risk of several cancers.

**Methods:**

To examine the role of the lectin complement pathway, we combined data from prospectively collected cohorts with available DNA specimens. Using a nested case-control design, we genotyped 85 single nucleotide polymorphisms (SNPs) in 9 genes in the lectin complement pathway and 3 additional SNPs in *MBL2* were tested post hoc). Initial SNPs were selected using tagging SNPs for haplotypes; the second group of SNPs for *MBL2* was selected based on functional SNPs related to phenotype. Associations were examined using logistic regression analysis. All statistical tests were two-sided. Nominal p-values are presented and are not corrected for multiple comparisons.

**Results:**

A total of 143 glioma cases and 419 controls were available for this analysis. Statistically significant associations were observed for two SNPs in the mannose-binding lectin 2 (*ML2*) gene and risk of glioma (rs1982266 and rs1800450, test for trend *p* = 0.003 and *p* = 0.04, respectively, using the additive model). One of these SNPs, rs1800450, was associated with a 58% increase in glioma risk among those carrying one or two mutated alleles (odds ratio = 1.58, 95% confidence interval = 0.99–2.54), compared to those homozygous for the wild type allele.

**Conclusions:**

Overall, our findings suggest that MBL may play a role in the etiology of glioma. Future studies are needed to confirm these findings which may be due to chance, and if reproduced, to determine mechanisms that link glioma pathogenesis with the MBL complement pathway.

## Introduction

Gliomas comprise 32% of all brain tumors and 80% of all malignant brain tumors [1] and encompass a spectrum of histologies that include low grade gliomas, anaplasic astrocytomas and glioblastomas (GBM). Fewer than 4.5% of patients diagnosed with GBMs, the most common glioma subtype (60%), are alive five years post-diagnosis [1]. There are no established environmental risk factors for gliomas other than ionizing radiation [2]. Several rare genetic syndromes, such as neurofibromatosis type I (NF1) increase the risk of brain tumors, and several genetic regions have been identified in genome-wide association studies (GWAS) to be related to risk of glioma [3], [4], [5]. However, while necessary when conducting agnostic analyses, over correcting for multiple comparisons can result in missed genetic associations that are etiologically important. This is particularly true in candidate gene analyses, where SNPs are selected in specific genes and prior probability for associations is hard to estimate, but in any event is higher than when using unselected polymorphisms.

Candidate-gene approaches can be used to interrogate the role of genetic variations in hypothesized etiologic pathways. The complement pathway has recently been identified as playing a role in oncogenesis [6]. Mannose-binding lectin 2 gene (*MBL2*), which is located on chromosome 10 and codes for a 32-kDa plasma glycoprotein, is involved in the innate immune response [7]. MBL2 binds to sugars on pathogens, leading to their destruction by opsonisation through the activation of the lectin complement pathway. MBL can also induce uptake of apoptotic cells and cell debris into macrophages [8], [9]. Individuals with low functional levels of MBL are more susceptible to bacteria, viral, and fungal infections [7]. A number of polymorphisms in *MBL2* have been associated with MBL serum level [7]. The first polymorphism discovered was a point mutation in codon 54 of exon 1 (rs1800450) which results in an amino acid exchange (glycine is replaced by aspartic acid due to a missense mutation) and in low serum levels of MBL [10]. Subsequently, numerous additional SNPs have been identified that influence serum levels and different haplotypes (often described as ‘secretor’ haplotypes) have been directly linked to serum levels [7], [11].

To date, five studies have examined the association between *MBL2* polymorphisms and risk of cancer, including breast cancer [12], stomach cancer [13], [14], colon cancer [15] and cervical cancer [16]. In these studies, *MBL2* polymorphisms resulting in lower serum levels have been associated with higher risk of cancer. In contrast, a study of lung cancer reported improved survival with the polymorphisms predicting low serum MBL levels [17]. No associations were reported with the *MBL2* gene and colon cancer survival [18].

SNPs in *MBL2* and the complement pathway have not been examined in relation to risk of glioma. Thus, we examined genes in this pathway using a candidate-gene approach and by pooling data from three prospective cohort studies.

## Methods

### Ethics Statement

This investigation was approved by the Institutional Review Board the Brigham and Women’s Hospital and/or the Harvard School of Public Health. All participants provided informed written consent.

### Study Populations

We identified cases and controls from three large US prospective cohort studies: the Physicians’ Health Study (PHS) [19], the Nurses’ Health Study (NHS) [20], and the Health Professionals Follow-up Study (HPFS) [21]. In brief, the PHS, a randomized controlled trial of 22,071 male physicians, aged 40–84 years, was initiated in 1982 to assess the efficacy of aspirin in reducing cardiovascular disease mortality and the efficacy of beta-carotene in reducing overall cancer incidence; the trial ended and is currently followed as an observational cohort. The NHS, initiated in 1976, is a prospective study of 121,700 female US registered nurses, aged 30–55 years examining risk factors for cancer, cardiovascular disease, and diabetes. The HPFS includes 51,529 US male health professionals, aged 40–75 years, who were recruited in 1986 and have been followed over time to examine lifestyle and dietary associations with major disease outcomes. As cases and controls were selected from the same base population as defined by the cohorts, the potential for population stratification is limited. A number of GWAS studies have been carried out in nested case-control studies from these cohorts, all showing little to no population stratification [22], [23]. Moreover, we restricted our analyses to self-reported Caucasian individuals to reduce potential population stratification.

### Blood Collection

Blood samples were collected from 14,916 PHS participants as part of the initial trial, by sending venipuncture kits to the 22,071 participants (return rate 68%) between August 1982 and December 1984. Samples were centrifuged, aliquoted, and stored in liquid nitrogen freezers at −80°C as either whole blood or plasma. For the NHS, blood samples were collected from 32,826 cohort subjects from May 1989 through September 1990. Blood collection kits were sent to 59,923 nurses who had indicated that they would be willing to send a blood sample. Upon arrival in the laboratory, the blood samples were centrifuged, and the samples were divided into eight aliquots and stored in liquid nitrogen freezers. In 1993 and 1994, blood specimens were collected from men in HPFS. A venipuncture kit was sent to all men who agreed to provide blood, 18,225 of whom returned the specimens on ice using an overnight courier. Multiple aliquots of plasma, buffy coat for DNA analyses, and red cells were stored in liquid nitrogen freezers for use in future assays.

### Case Ascertainment and Control Selection

Cancers are reported by participants in annual (PHS) or biennial (HFPS and NHS) follow-up questionnaires. Medical records and pathology reports were obtained from hospitals after permission was received from subjects or for deceased cases identified through the National Death Index (NDI) or other sources from next-of-kin. Deaths are usually reported by family members or by the postal service in response to mailed questionnaires. In addition, the NDI was searched biennially for non-respondents, and this method has been shown to have a sensitivity of 98% [24]. Approximately 88% of potential case subjects (self-reported or deceased cases with glioma) were subsequently confirmed with medical, pathology, cancer registry data or death certificate. For the present analysis, we included cases with any type of glioma brain tumor: astrocytoma, glioblastoma, oligodendroglioma, ependymoma, and mixed glioma subtypes.

All confirmed incident glioma cases, which provided blood samples at baseline, were selected for this study. For each case, three control subjects were identified among the cohort participants who returned blood samples, who did not have cancer, and who were alive at the time the matched case was diagnosed. Controls were chosen at random and matched with each case on year of birth, cohort (which automatically matches the sex, because each cohort consists of either men only or women only), month of blood sample collection, and race background. In the PHS cohort, controls were also matched to each case based on their randomly-assigned intervention group.

### Candidate Gene and SNP Selection

DNA extractions were completed at the Channing Laboratory (Boston, MA) and shipped to Imperial College in London where a custom Illumina assay chip (Illumina, Inc., San Diego, CA) was designed to amplify 384 SNPs distributed among approximately 40 genes. Most of the genes included in the assay were chosen because they have either been associated with allergy and atopy. In addition to the allergy genes, we included SNPs on 9 genes in the complement pathway (total 85 SNPs) [6]; gene (number of SNPs tested): *C1QA* (4), *C1QB* (7), *C1QC* (3), *C2* (2), *C3* (23), *C5* (23), *C5AR1* (3), *CFB* (10), and *MBL2* (7). Three additional functional SNPs in MBL2 were selected and tested post-hoc (see below). For each gene, we chose a partially redundant set of haplotype tagging SNPs, using the HapMap data phaseIII/Rel #2 for the CEU population. Partial redundancy was used to protect against information loss in case one haplotype tagging SNP failed to amplify. The CEU population was chosen as the reference population for SNP tagging as the subjects in this study were Caucasians.

### Genotyping and Quality Control

DNA was obtained from all patients, and DNA concentrations were checked using Quant-iT™ PicoGreen® dsDNA reagent (Invitrogen, Carlsbad, CA) and normalized to 50 ng/µl before genotyping. A total of 250 ng DNA was used for the Illumina GoldenGate Assay (Illumina, Inc., San Diego, CA), which was performed according to the manufacturer’s standard three-day protocol. Each genotyping plate was prepared with both cases and controls to avoid plate-specific bias. Illumina clustering was performed on the raw data using Illumina’s Genome Studio software version 2009.1. All 384 SNPs were also manually inspected to make sure the clusters were correctly called after sorting via a statistical score called Gentrain. This score varies from 1 to 0 and is based on the shape of the clusters and their relative distance to each other. The clusters were manually called for SNPs that were not correctly called by the software. All SNPs that could not be called (due to overlapping clusters, more than three clusters, or low intensity) were zeroed and excluded (n = 16, 4.2%). At least one replicate was included per plate for internal quality control, giving a total of 9 replicates across plates. The reproducibility frequency for the 368 SNPs (after removing the SNPs that could not be called) genotyped was 99.8%. A total of 5 HapMap Trios were also used as internal quality controls, and the data gave an overall parent-parent-child heritability percentage of 99.6%. Individuals with call rate of <75% (n = 20) were excluded.

In the overall Illumina plate results, we observed strong associations for SNPs previously associated with glioma (i.e., SNPs in genes *TERT, RTEL1* and *PHLDB1*, included on plate to serve as controls). In addition, we observed strong associations for three *MBL2* SNPs (rs1982266, rs4935047, rs10824793; which are in high LD). However, after reviewing the literature, we discovered that our selected tagging SNPs in *MLB2* did not include key SNPs that contributed to the well-studied secretor *MLB2* haplotype to provide information on serum levels of MLB2. Thus, we genotyped 5 additional SNPs (rs1800450, rs1800451, rs5030737, rs7096206, rs11003125) using TaqMan (Applied Biosystems) according to manufacturer’s instructions. The following Applied Biosystem’s assay IDs were used to order four of the SNP assays: rs1800450, assay ID: C_2336609_20; rs7096206, assay ID: C_27858274_10; rs1800451, assay ID: C_2336608_20; rs5030737, assay ID: C_2336610_10. A custom assay by design was ordered for SNP rs11003125 (Forward Primer Sequence: GGAGTTTGCTTCCCCTTGGT, Reverse Primer Sequence: GGGCCAACGTAGTAAGAAATTTCCA, Reporter 1 Dye: VIC, Reporter 1 Sequence: CAAGCCTGTGTAAAAC, Reporter 2 Dye: FAM, Reporter 2 sequence: CAAGCCTGTCTAAAAC). Unfortunately, 3 of the 5 SNPs that have known impact on MBL2 serum levels have rare alleles with MAF<0.05; data for two of the SNPs (rs1800451 and rs5030737) were monomorphic and could not be included in the data analyses. Deviations of the observed genotype frequencies from the Hardy-Weinberg equilibrium (HWE) were evaluated and all three SNPs (rs1800450, rs7096206 and rs11003125) were in HWE (p>0.05). Genotyping success rate for rs1800450 was 83%, rs7096206 was 97% and rs11003125 was 84%. A total of 8 replicates were used for internal quality control assessment and showed >99% concordance for each SNP assay.

### Statistical Analyses

Departures from Hardy-Weinberg equilibrium for each SNP were determined using a χ^2^ test. We used unconditional logistic regression analyses to estimate odd ratios (OR) and corresponding 95% confidence intervals (95% CI) for individual SNPs and risk of glioma, controlling for age and cohort (conditional models resulted in similar OR estimates). Associations were evaluated using co-dominant models unless the minor allele frequency was less than 0.05 in controls (in which case the dominant model was used). Test for trends were estimated using the Cochran-Armitage test. Haplotype analyses were constructed for *MBL2* using 10 SNPs in two blocks previously identified using HapMap and including the SNPs that were identified from the literature post-hoc. The haplotype-specific odds ratios were estimated from genotype data on unrelated cases and controls using unconditional logistic regression. We used an “expectation substitution” approach [25], [26], which treats expected haplotype scores (calculated under a user-specified inheritance model) as observed covariates in a standard unconditional logistic analysis. All tests of statistical significance were two-sided, and *P* values less than.05 were considered statistically significant. All analyses were performed using SAS 9.1 (SAS Inc., Cary, NC).

## Results

After filtering poor performing samples, the final analysis included 143 cases and 419 controls. The mean age of the cases at blood draw and time of diagnosis was 59 and 68 years, respectively (Table 1). Controls were matched on age at blood draw, cohort (which also matches for sex), and month of blood sample collection.

**Table 1:** Characteristics of cases and controls from three prospective cohort studies*.

	Cases		Controls	
	No.	Mean (SD)or %	No.	Mean (SD)or %
Age at blood draw, y	143	58.9 (8.9)	419	58.6 (8.9)
Sex, males	143	66%	419	63%
Age at diagnosis, y	143	68.3 (9.3)	**–**	**–**

* The blood draw period of all cohorts were as follows: NHS, 1989–1990; HPFS, 1993–1994; PHS, 1982–1984. SD = standard deviation; NHS = Nurses’ Health Study; HPFS = Health Professionals Follow-up Study; PHS = Physicians’ Health Study; **–** = controls do not have an age at diagnosis as they did not develop cancer.

We observed an association with a tagging SNP on *MBL2* (rs1982266; OR = 0.36, 95% CI = 0.19–0.66 for homozygous recessive compared to homozygous dominant, test for trend *p* = 0.003) and glioma risk in our preliminary analysis of SNPs selected from HapMap that cover 10 genes in the complement pathway. As we could not determine the secretor phenotype from this SNP, we reviewed the literature and selected new SNP variants that had been linked to serum MBL2 levels in prior studies [7], [11]; all SNP results for *MBL2* are presented in Table 2. SNP rs1800450 represents a structural mutation in exon 1 of *MBL2* at codon 54 (historically referred to allele as “*B”* and resulting in an amino acid exchange from Gly to Asp); those with missense mutation in allele *B* have low levels of serum MBL [7]. We observed a dose-dependent increase in risk with SNP rs1800450 (test for trend, *p* = 0.04). A 58% increase in risk of glioma was observed among those carrying one or two missense mutations in rs1800450 (odds ratio = 1.58, 95% confidence interval = 0.99–2.54), compared to those homozygous for the wild type allele.

**Table 2:** Association between *MBL2* SNPs and risk of glioma in 3 prospective cohort studies.

SNP	Cases	Controls	OR^a^	95% CI	*P*
	n (%)	n (%)			
rs10824792					
AA	40 (28.8)	139 (34.1)	1.0	ref.	
AG	73 (52.5)	195 (47.8)	1.30	(0.83–2.02)	
GG	26 (18.7)	74 (18.1)	1.22	(0.70–2.16)	0.39
rs11595876					
AA	120 (86.3)	372 (90.5)	1.0	ref.	
AG	18 (12.9)	35 (8.5)	1.60	(0.87–2.92)	
GG	1 (0.7)	0. 4 (1.0)	0.73	(0.08–6.65)	0.26
rs930508					
CC	94 (68.1)	262 (63.7)	1.0	ref.	
CG	40 (29.0)	132 (32.1)	0.86	(0.56–1.32)	
GG	4 (2.9)	17 (4.1)	0.65	(0.21–1.98)	0.34
rs1838066					
AA	50 (35.7)	176 (42.8)	1.0	ref.	
AG	72 (51.4)	175 (42.6)	1.45	(0.95–2.20)	
GG	18 (12.9)	60 (14.6)	1.05	(0.57–1.95)	0.43
rs1982266^b^					
GG	53 (37.9)	113 (27.8)	1.0	ref.	
GA	70 (50.0)	193 (47.2)	0.78	(0.51–1.20)	
AA	17 (12.1)	102 (25.1)	0.36	(0.20–0.66)	0.003
rs1800450^c^					
CC	81 (69.8)	271 (78.6)	1.0	ref.	
CT	32 (27.6)	71 (20.6)	1.51	(0.93–2.45)	
TT	3 (2.6)	3 (0.9)	3.44	(0.68–17.4)	0.04
rs7096206^d^					
CC	86 (61.9)	238 (59.5)	1.0	ref.	
CG	47 (33.8)	139 (34.8)	0.95	(0.63–1.44)	
GG	6 (4.3)	23 (5.8)	0.74	(0.29–1.88)	0.58
rs11003125^e^					
CC	50 (41.3)	146 (42.0)	1.0	ref.	
CG	55 (45.5)	146 (42.0)	1.09	(0.70–1.71)	
GG	16 (13.2)	56 (16.1)	0.82	(0.43–1.56)	0.72

a Odds ratio adjusted for age and study (cohort; sex).

b rs4935047 and rs10824793 are in complete LD with rs1982266 and thus results are not shown.

c Also known as Ex1-27G>A; A/B.

d Also known as −289 G>C; X/Y.

e Also known as −618 G>A; H/L.

When restricting the analysis to glioblastomas (n = 98), the association was similar for SNP rs1982266 (OR = 1.56, 95% CI = 1.13–2.16, in the dominant model; *p*-trend using additive model = 0.006), but none of the remaining *MBL2* SNPs were individually significantly related to risk.

We conducted a haplotype analysis using tagging SNPs (Table 3); while we cannot directly relate blood MBL phenotype to the haplotypes using our tagging SNPs, a statistically significant association was observed for one of the haplotypes that was not explained by any of the individual SNPs. The haplotype analysis for glioblastoma was very similar to the overall results for glioma risk (data not shown).

**Table 3:** *MBL2* haplotypes associated with risk of glioma in 3 prospective studies.

Haplotype^a^	Cases	Controls	OR^b^	95% CI
AACGAGGCCG	75.9(26.9%)	238.44 (28.7%)	1.0	Ref.
AACAGAACCC	42.9 (15.2%)	155.5 (18.7%)	0.87	0.58–1.32
GAGAGAACGC	46.4 (16.5%)	140.2 (16.9%)	1.04	0.68–1.59
GACAAGGTCC	29.3 (10.4%)	86.0 (10.3%)	1.01	0.61–1.67
AGCGAGGCCG	16.5 (5.8%)	41.4 (5.0%)	1.24	0.68–2.28
GACAAGGCCC	18.4 (6.5%)	30.0 (3.6%)	2.10	1.05–4.19
GACAGAACGC	12.0 (4.3%)	31.5 (3.8%)	1.27	0.60–2.67
GACAGAACCC	10.9 (3.9%)	31.6 (3.8%)	1.06	0.50–2.27
AACGAGGCCC	7.1 (2.5%)	16.7 (2.0%)	1.36	0.49–3.79
AACAAGGTCC	5.4 (1.9%)	11.1 (1.3%)	1.82	0.55–6.08

*P* of LRT test = 0.30.

*P* for global test = 0.66.

a Haplotype defined as (in this order): rs10824792; rs11595876; rs930508; rs1838066; rs10824793; rs4935047;rs1982266; rs1800450; rs7096206; rs11003125.

b Adjusted for age and study (cohort; sex).

Except for the *MBL2* gene, we observed no robust associations for the complement genes included in our SNP panel (Table S1) when testing single SNPs or haplotypes for these genes. Borderline statistically significant associations were observed for two SNPs with rare variants (allele frequency <0.05); *p*-values for dominant model = 0.04 for rs36221133 (*C2* gene), and 0.01 for rs4467185 (*C5AR1* gene).

## Discussion

In this prospective study combining data from three large cohorts, we observed significant associations between SNPs in *MBL2* gene and risk of glioma. SNPs in other genes in the lectin complement pathway were not related to glioma risk.

MBL levels have been associated with respiratory infections, diarrheal disease, atopy and failure to thrive during infancy [7], [11]. Several polymorphisms in the *MBL2* gene have been linked to serum MBL and these have been widely studied [11]. Point mutations in codon 54 (allele *B*), codon 57 (allele *C*), and codon 52 (allele *D*) of exon 1 of the *MBL2* gene result in very low serum levels of MBL (for homozygous or heterozygous alleles *B* and *C*, and to a lesser degree with allele *D*) [11]. Other polymorphisms have been associated with MBL serum levels and together these SNPs form haplotypes that explain variations in MBL serum levels [11].

In this study, a mutation in codon 54 (rs1800450) was associated with a higher risk of glioma which might suggest that low serum MBL levels may increase risk of glioma. Another SNP (rs1982266) in the *MBL2* gene was strongly associated with risk; this SNP has not been previously studied and is not in strong LD (R^2^ = 0.125) with the codon 54 mutation. More studies are needed to evaluate how variants of rs1982266 may influence serum MBL levels in order to determine if, and how, MBL2 serum levels are associated with glioma risk.

To date, several studies have examined the relationship between polymorphisms in the *MBL2* gene and cancer risk. In one study on gastric cancer, a strong increase in risk was observed with exon 1 allele *D* of MBL2 gene (haplotype analysis for HYD, compared to HYA: OR = 1.9, 95% CI = 1.1–3.2), although no associations were found with alleles *B* or *C* [13]. In another study of stomach cancer, no overall association was observed with the *MBL2* gene, although only codon 54 was measured [14], and this SNP was not associated with stomach cancer in the study by Baccarelli *et al* [13]. In a recent study examining a large number of SNPs in the *MBL2* gene, including SNPs in the 3′UTR region, haplotypes associated with low MBL serum levels were associated with elevated risk of colon cancer in African-Americans but not Caucasians [15]. Similar associations were also noted between *MBL2* haplotypes and breast cancer risk among African-American women, but not Caucasians [12].

There has also been interest to determine whether *MBL2* polymorphisms can influence rates of viral infections related to cancer, including human papillomavirus (HPV) infection [16], [27], hepatitis C virus [28] and BK virus coinfection in HPV-positive cervical lesions [29]. These studies have reported mixed results.

The impact of MBL serum levels on cancer risk might be directly linked to its ability to bind apoptotic or necrotic cells and facilitate macrophage uptake without activating the complement pathway; recent *in vitro* studies show that MBL may act independently of the complement cascade by facilitating the ingestion of apoptotic cells by human macrophages through macropinocytosis [8], [30]. The role of the immune response and inflammatory response in relation to carcinogenesis is complex [31] and the role of MBL on cancer risk requires further study.

A limitation of our study is the small sample size, despite pooling data from 3 large cohort studies; future studies will have to reproduce our findings. Another limitation is the genotype failure for SNP rs1800451 (allele *C*); data on this SNP might have provided more information on genetic variation that affect serum levels of MBL. We did not directly measure MBL levels in plasma (as it was not part of the original aims of the funded cohort study), accordingly we could not directly examine the relation between haplotypes and MBL serum levels; however, the literature on the relation between the polymorphisms in this gene and associated phenotypes is well documented [7], [11].

To our knowledge, this is the first study to examine *MBL2* polymorphisms and risk of glioma. We observed a strong association between two *MBL2* polymorphisms which are not in high linkage disequilibrium and risk of glioma. While our results will need to be confirmed in a larger sample, they may provide a novel and exciting area for future research on glioma pathogenesis. The role of the immune system in glioma etiology is critical, as demonstrated by the numerous consistent findings for allergies and IgE [32], and needs further elucidation.

## Supporting Information

Table S1 Name of complement genes and SNPs examined in this study.
(DOCX)
