# Supplementary material for: Mannose-Binding Lectin 2 Gene and Risk of Adult Glioma
Source: PLoS One. 2013 Apr 18;8(4):e61117. doi: 10.1371/journal.pone.0061117 (PMC3630225; doi:10.1371/journal.pone.0061117)
Supplement: Table S1 — Name of complement genes and SNPs examined in this study. (DOCX) [file pone.0061117.s001.docx]

**Supplement Table 1. Complement genes and SNPs examined in this study^.*^**

| SNP |  | Chromosome | Position | Gene sympbol | Location |
| --- | --- | --- | --- | --- | --- |
| rs2935542 | [A/G] | 1 | 22836057 | C1QA | intron |
| rs12740591 | [A/G] | 1 | 22837589 | C1QA | intron |
| rs292001 | [T/C] | 1 | 22837544 | C1QA | intron |
| rs665691 | [G/C] | 1 | 22832940 | C1QA | flanking_5UTR |
| rs12722729 | [A/G] | 1 | 22854652 | C1QB | intron |
| rs623183 | [T/C] | 1 | 22859217 | C1QB | intron |
| rs629409 | [T/C] | 1 | 22859324 | C1QB | intron |
| rs12756603 | [T/C] | 1 | 22854462 | C1QB | intron |
| rs291989 | [T/C] | 1 | 22852485 | C1QB | intron |
| rs291985 | [A/C] | 1 | 22853525 | C1QB | intron |
| rs7549747 | [A/G] | 1 | 22869157 | C1QB | flanking_3UTR |
| rs294185 | [A/G] | 1 | 22844478 | C1QC | intron |
| rs294183 | [A/G] | 1 | 22844684 | C1QC | intron |
| rs12404537 | [A/G] | 1 | 22841954 | C1QC | flanking_5UTR |
| rs1042663 | [A/G] | 6 | 32013108 | C2 | coding |
| rs36221133^**^ | [T/C] | 6 | 32020501 | C2 | coding |
| rs11569501 | [A/G] | 19 | 6642630 | C3 | intron |
| rs406514 | [A/G] | 19 | 6653597 | C3 | intron |
| rs11569429 | [T/C] | 19 | 6660073 | C3 | intron |
| rs7257062 | [T/C] | 19 | 6636944 | C3 | intron |
| rs11672613 | [A/G] | 19 | 6656245 | C3 | intron |
| rs344550 | [C/G] | 19 | 6633952 | C3 | intron |
| rs4807895 | [A/G] | 19 | 6662957 | C3 | intron |
| rs2230199 | [C/G] | 19 | 6669386 | C3 | coding |
| rs2241393 | [G/C] | 19 | 6636303 | C3 | intron |
| rs2241392 | [G/C] | 19 | 6636982 | C3 | intron |
| rs2241394 | [G/C] | 19 | 6636229 | C3 | intron |
| rs344555 | [T/C] | 19 | 6630359 | C3 | intron |
| rs344548 | [C/G] | 19 | 6636816 | C3 | intron |
| rs11569523 | [A/G] | 19 | 6640041 | C3 | intron |
| rs8112351 | [A/G] | 19 | 6657297 | C3 | intron |
| rs237554 | [A/G] | 19 | 6637658 | C3 | intron |
| rs11569515 | [A/C] | 19 | 6641243 | C3 | intron |
| rs2287846 | [C/G] | 19 | 6647556 | C3 | intron |
| rs7951 | [A/G] | 19 | 6632990 | C3 | coding |
| rs432823 | [T/C] | 19 | 6653245 | C3 | intron |
| rs344540 | [A/G] | 19 | 6638767 | C3 | intron |
| rs2277984 | [T/C] | 19 | 6630510 | C3 | intron |
| rs11569508 | [A/G] | 19 | 6641745 | C3 | intron |
| rs7867876 | [T/C] | 9 | 122763354 | C5 | intron |
| rs1978270 | [T/A] | 9 | 122802496 | C5 | intron |
| rs12237868 | [T/A] | 9 | 122788575 | C5 | intron |
| rs16910280 | [T/C] | 9 | 122844596 | C5 | intron |
| rs7027797 | [A/G] | 9 | 122811619 | C5 | intron |
| rs10818494 | [T/C] | 9 | 122786258 | C5 | intron |
| rs10818500 | [T/C] | 9 | 122850703 | C5 | intron |
| rs7046909 | [T/C] | 9 | 122803700 | C5 | intron |
| rs10514838 | [A/G] | 9 | 122786616 | C5 | intron |
| rs7861142 | [T/C] | 9 | 122786619 | C5 | intron |
| rs7872790 | [A/T] | 9 | 122787973 | C5 | intron |
| rs2269067 | [C/G] | 9 | 122776860 | C5 | intron |
| rs992670 | [T/C] | 9 | 122821590 | C5 | intron |
| rs2159776 | [A/G] | 9 | 122795980 | C5 | intron |
| rs3815467 | [T/C] | 9 | 122832726 | C5 | intron |
| rs2241004 | [A/G] | 9 | 122821958 | C5 | intron |
| rs2300934 | [T/G] | 9 | 122848783 | C5 | intron |
| rs10733650 | [A/G] | 9 | 122824318 | C5 | intron |
| rs12004487 | [A/G] | 9 | 122756501 | C5 | intron |
| rs41258306 | [T/C] | 9 | 122755923 | C5 | coding |
| rs17612 | [A/C] | 9 | 122765746 | C5 | coding |
| rs12237774 | [T/C] | 9 | 122765791 | C5 | coding |
| rs25681 | [A/G] | 9 | 122819825 | C5 | coding |
| rs4427917 | [C/G] | 19 | 52507554 | C5AR1 | intron |
| rs4577202 | [A/G] | 19 | 52507761 | C5AR1 | intron |
| rs4467185^**^ | [A/G] | 19 | 52514877 | C5AR1 | coding |
| rs4151657 | [A/G] | 6 | 32025518 | CFB | intron |
| rs537160 | [T/C] | 6 | 32024378 | CFB | intron |
| rs2072633 | [T/C] | 6 | 32027556 | CFB | intron |
| rs1270942 | [T/C] | 6 | 32026838 | CFB | intron |
| rs4151651 | [A/G] | 6 | 32023592 | CFB | coding |
| rs4151671 | [T/C] | 6 | 32026881 | CFB | intron |
| rs4151672 | [T/C] | 6 | 32027808 | CFB | 3UTR |
| rs4151670 | [A/G] | 6 | 32023510 | CFB | coding |
| rs2072634 | [A/G] | 6 | 32025269 | CFB | coding |
| rs4151659 | [A/G] | 6 | 32026442 | CFB | coding |

^*^Some SNPs are in high LD and were selected to allow for redundancy in case of SNP failure.

^**^*p*-values for dominant model were <0.05.
